# Impact of Smartphone Apps on Reperfusion Times and Clinical Outcomes in Acute ST-Segment Elevation Myocardial Infarction: Systematic Review and Meta-Analysis

**DOI:** 10.2196/66605

**Published:** 2025-08-25

**Authors:** William Gibson, Dawoud Al Kindi, Elie Akl, Kshitij Badal Dandona, Jean-Philippe Pelletier, Nicolo Piazza, Ali Zgheib, Giuseppe Martucci, Marco Spaziano

**Affiliations:** 1 Department of Cardiology, Royal Victoria Hospital, McGill University Health Centre, 1001 Decarie Blvd, Montreal, QC, H4A 3J1, Canada, 1 5146072766

**Keywords:** door-to-balloon time, ST-elevation myocardial infarction, STEMI, cardiology, cardiac, cardiologists, Telemedicine, Telehealth, tele-medicine, tele-health, virtual care, virtual health, virtual medicine, mHealth, mobile health, mobile applications, mobile apps, apps, smartphones, applications, digital, digital health, digital technology, digital interventions

## Abstract

**Background:**

Smartphone- and tablet-based apps have been increasingly used in the management of acute ST-segment elevation myocardial infarction (STEMI), with the goal of enhancing care efficiency. These apps facilitate improved team coordination through a single platform, enabling secure sharing of clinical data, arrival times, and enabling data storage and processing capabilities. The potential of these technologies to reduce reperfusion times and improve both clinical and process outcomes, compared to traditional communication methods, is promising.

**Objective:**

This study aimed to evaluate the effectiveness of smartphone-based interventions in reducing door-to-balloon (D2B) time, first medical contact-to-balloon (FMC2B) time, mortality, and false activation rates in STEMI care pathways.

**Methods:**

This review followed the PRISMA guidelines and the PICO framework for eligibility criteria. Studies were included if they compared smartphone- or tablet-enabled interventions with usual care for STEMI management, focusing on D2B time, FMC2B time, short-term mortality, and false activation rates. A systematic literature search was conducted across MEDLINE, Embase, and Google Scholar for studies published between 2008 and 2024. Studies using purpose-built software or commercially available instant messaging apps that enabled digital ECG transfer and real-time communication between providers were included. The protocol was registered with PROSPERO (CRD42023481024). Data synthesis was performed using SPSS (IBM) with random-effects meta-analysis for continuous and binary outcomes.

**Results:**

A total of 903 articles were identified after removing duplicates, and 21 studies, involving 3267 patients, were included. Studies varied in design: 14 being retrospective and 7 prospective, conducted across 12 countries. Thirteen studies evaluated dedicated apps, and 8 used instant messaging platforms such as WhatsApp (Meta Platforms Inc) and WeChat (Tencent Holdings Ltd). The primary outcome, D2B time, showed a significant reduction in the intervention group (mean difference –19.11 mins, 95% CI –26.22 to −12.00; *P*<.01), with substantial heterogeneity (*I*²=89%). A similar reduction in FMC2B time was observed (mean difference −19.85 minutes, 95% CI −29.45 to −20.25; *P*=.01). Subgroup analysis indicated a more pronounced reduction in D2B time in low-income countries compared to high-income countries. There were no significant differences regarding short-term mortality (risk difference −0.03, 95% CI −0.07 to 0.01; *P*=.10). False activation rates were evaluated in 7 studies, with varying results, but no pooled analysis was feasible due to differences in definitions and study design. The health care setting (ie, low- or high-income countries) was the most significant factor contributing to the observed heterogeneity in the meta-regression analysis.

**Conclusions:**

Smartphone-based interventions significantly reduce reperfusion times in STEMI care pathways. Digital technology can improve the efficiency of STEMI management, particularly with lower-resource health care infrastructure. Future studies should explore the impact on long-term outcomes and investigate regional differences in treatment effects.

## Introduction

ST-segment elevation myocardial infarction (STEMI) is a time-sensitive medical emergency in which prompt diagnosis and management is integral to minimizing total ischemic time and reducing morbidity and mortality [1]. Ischemic time is influenced by a number of elements, and delays may result from patient, emergency services, or systemic factors, which can further vary depending on the mode of patient presentation (ie, direct presentation to a PCI-capable ED, to a non-PCI center ED, or via emergency medical services [EMS].)" A number of time goals to achieving reperfusion are recommended by societal best practice guidelines internationally [2], and there is evidence to suggest an association between shorter door-to-balloon (D2B) times and improved survival outcomes. One pooled analysis including 300,000 patients from 32 prospective cohort studies demonstrated an association between longer D2B times (>90 mins) and increased short-term mortality [3]. Furthermore, shorter D2B times have been associated with lower hospital readmission rates and greater economic efficiency.

Achieving reperfusion within these targets requires streamlined patient transition, achieved by adequate coordination between the multidisciplinary systems involved in the chain of care and promoted by effective communication. Furthermore, coordinated efforts to collect, access, and interpret data efficiently are integral to improving the quality of these systems on an ongoing basis through continuous clinical audit. While patient delay is multifactorial and necessitates public health measures to enhance awareness of symptoms to reduce the time to the “first call for help,” system delays can be amenable to improvements by organizational means [4]. Advancements in telemedicine technologies have improved the efficiency of acute STEMI care in recent years, primarily through the transmission of prehospital electrocardiograms, leading to earlier activation of the catheterization laboratory (cath lab) and mobilization of the STEMI team. For example, prehospital electrocardiogram (ECG) transmission was associated with a 40% relative reduction in time to treatment in one meta-analysis, including 11 nonrandomized studies [5], while this effect was mirrored in a more recent analysis which further demonstrated an association between the implementation of a telemedicine strategy in acute STEMI and a reduction in mortality compared with usual care [6]. Notably, these reviews primarily included outdated telecommunication methods such as telephone and fax. However, a recent meta-analysis of nonrandomized studies, explored the impact of exclusively digital prehospital ECG transmission methods on reperfusion times and mortality and noted a significant reduction in door-to-device times (−33.3 mins) and in all-cause mortality (hazard ratio 0.53, 95% CI 0.40‐0.69), highlighting the potential benefits of digital technology in this setting.

Nowadays, mobile devices are ubiquitous and an essential tool for health care practitioners in their day-to-day practice, with accompanying technological features including Wi-Fi, GPS, Bluetooth pairing, cameras, microphones, sensors, and cloud storage [7,8]. These capabilities enhance the potential use of mobile health (mHealth) apps to enhance care coordination in acute STEMI care. A number of apps have emerged in this domain, using tailor-built platforms as well as commercially available, nondedicated instant messaging apps (eg WhatsApp [Meta Platforms Inc] [9]. In comparison to instant messaging platforms, dedicated apps provide a number of added functions beyond a communication channel alone. First, they allow for notification of each individual STEMI team members’ acknowledgment of the incoming case which can foster more rapid and effective mobilisation of the team. They may further enable GPS and live time tracking of the patient relative to reperfusion targets, with integrated push alerts in situations where time to reperfusion may be too long to favor pPCI, and fibrinolytic therapy may be warranted instead. Moreover, they may provide a single interface for the entry of automated time stamped data which can be accessed easily, promoting improved efficiency of clinical auditing. Of further relevance, these technologies can be tailored to comply with data regulations ensuring confidentiality of storage and transmission of protected health information between health care providers [10-12]. At the time of writing, and to the best of our knowledge, there have been no reviews published exploring the effects of these technologies on outcomes in acute STEMI. This systematic review and meta-analysis aims to (1) evaluate the impact of smartphone-based STEMI coordination apps on reperfusion time targets and mortality in acute care settings, (2) identify the key features of available apps that may contribute to improved clinical efficiency and patient outcomes, and (3) compare the effects of dedicated versus nondedicated platforms, assessing their potential advantages and limitations in streamlining STEMI care. By synthesizing the current evidence, this review seeks to provide insights into the role of mHealth technologies in optimizing acute STEMI pathways and informing future advancements in digital health care solutions.

## Methods

### Selection and Eligibility Criteria

The review was conducted in accordance with the PRISMA (Preferred Reporting Items for Systematic Reviews and Meta-Analyses) guidelines; the prespecified eligibility criteria and search strategy were structured using the PICO (population, people, patient, or problem), intervention, comparison and outcome template as displayed in Table 1. The study protocol was registered with PROSPERO (the International Prospective Register of Systematic Reviews; CRD42023481024.) Studies were included if they had a comparative study design and compared a strategy of smartphone or tablet-enabled technology as outlined in the eligibility criteria combined with usual care versus traditional methods of communication (eg, via telephone; review of STEMI ECG in person by STEMI team) for the diagnosis and management of patients with STEMI. The coprimary outcome was the difference in reperfusion times including door-to-balloon time and first medical contact-to-balloon (FMC2B) time; D2B time was defined as the time from arrival at the pPCI center to the deployment of a balloon within the infarct-related coronary artery, while FMC2B time refers to the time elapsed from first contact with a health care professional in the chain of care to the initial balloon inflation. Prespecified secondary outcomes of interest were the differences in short-term mortality and false activation rates between groups, in addition to the proportion of patients meeting guideline directed time targets, that is, D2B time ≤90 minutes or FMC2B time ≤120 minutes.

**Table 1.** PRISMA (Preferred Reporting Items for Systematic Reviews and Meta-Analyses) framework for study eligibility.

Criteria		Description
Population		Patients >18 years of age treated for suspected acute ST-segment elevation myocardial infarction in prehospital or hospital setting eligible to receive primary intervention (percutaneous coronary intervention or lysis where applicable).
Intervention		
	Inclusion criteria	Smartphone or tablet-enabled mobile apps. Used by health care provider. Cloud-based ECG^a^ transmission. Real-time communication between relevant health care providers or stakeholders involved in the chain of care in acute STEMI^b^.
	Exclusion criteria	Patient-centered app usage. Chronic care or postmyocardial infarction management. Nonmobile or cellular-enabled telemedicine. Posthoc communication (ie, not enabled in real time). Focus on qualitative or technical process–related outcomes.
Comparator		Patients treated for suspected acute STEMI using traditional communication methods (ie, nonmobile data sources or tablet app–based communication) between relevant stakeholders.
Outcomes		Reperfusion times (door to balloon and first medical contact to balloon), proportion of patients meeting targets (eg, D2B^c^ <90 min and FMC2B^d^ <120 min), false activation rate, and clinical events (eg, mortality and heart failure).

a ECG: electrocardiogram.

b STEMI: ST-segment elevation myocardial infarction.

c D2B: door to balloon.

d FMC2B: first medical contact to balloon time.

The types of digital technology studied in this review include purpose-built software applications and commercially available instant messaging apps into STEMI care pathways, granted the latter fulfilled the prespecified eligibility checklist, namely, digital ECG transfer and real time communication between providers at the point of care. In cases wherein duplicate studies were identified, those with the largest sample size and follow up duration were solely included.

A total of 2 investigators independently screened potential titles and abstracts and those with potential eligibility by either reviewer were selected for full-text review. Disagreements were subsequently resolved by a third reviewer. Efforts were made to contact corresponding authors as required where relevant data was not included in the published report. The article was subsequently excluded if a satisfactory response or the relevant data was not obtained. Attempts were made to contact all authors in relation to missing data and study design clarification via email to which 4 replies were received.

### Data Sources and Search Strategy

A systematic literature search was conducted across multiple databases, including MEDLINE, Embase and Google Scholar, to identify relevant studies published between January 1st, 2008, and February 1st, 2025. Textbox 1 details the structured search strategy used. The search was restricted to English-language studies, with exclusion of the term “rehab” to focus on studies evaluating acute care. To ensure comprehensive coverage, simultaneous searches of identified app names (eg, STENOA [STENOA Inc] and SCUNA [Mehergen) were performed using title-word searches.

Textbox 1. **Database search strategy.**

Search statement 1: “mhealth”.tw OR “mobile health”.tw OR “app”.tw OR “smartphone”.tw OR “smart phone”.tw OR “mobile phone”.tw OR “tablet”.tw OR “mobile application”.tw OR “m-health”.tw OR “mobile device”.tw OR “mobile app”.tw OR “mobile apps”.tw OR “cloud*”.tw

AND

Search statement 2: “STEMI”.tw OR “ST elevation myocardial infarction”.tw OR “door to balloon”.tw. OR “door-to-balloon”.tw OR “myocardial infarction”.tw OR “acute coronary syndrome”.tw OR “ACS”.tw

NOT “rehab*”.tw

### Data Extraction and Quality Assessment

A total of 2 investigators (WG and DAK) extracted data from each study according to a standardized protocol. Any disagreements were resolved by the third reviewer (MS). The methodological quality of each study included was assessed using the Newcastle-Ottawa Scale and 1 author (WG) assessed the quality of each study while a second author (DAK) reviewed these assessments in conjunction with the studies to support article inclusion. A third reviewer (MS) resolved any discordance.

### Quantitative Data Synthesis

All analyses were performed using the meta-analysis function within SPSS (IBM Corp) comparing app-based care with usual care with respect to D2B times, FMC2B, and the proportion of patients meeting reperfusion targets (<90 mins and<120 mins) with continuous outcome measurements (unstandardized mean difference in mins) and odds ratio (OR and 95% CI, accordingly). The risk difference was used for the secondary outcome of mortality. When data concerning the mean and variance were not available, efforts to obtain these were made by contacting the authors. Failing this, the mean and variance were estimated by using the median, IQR, sample size and/or reported CIs [13]. In cases where the SDs for the mean in both control and intervention groups were not given, these were derived from the mean difference and corresponding 95% CI and/or the *P* value, where available [14]. In situations where more than 1 control group was encountered, the group which bore more similarity to the intervention group was selected. For example, if the intervention group concerned patients transferred from a non-PCI capable hospital, compared to those transferred by EMS or direct ED presentations with whom no app was used, the EMS transfers were chosen as the control group for analysis. Owing to expected clinical heterogeneity, a random-effects model was used for pooling the results of included studies. Mean differences were calculated for continuous outcomes; pooled relative risks and pooled risk difference for binary outcomes, and calculated 95% CIs and 2-sided *P* values for each outcome. Cochran Q test, chi-square tests, and the *I*^2^ statistic were used to investigate statistical heterogeneity of the treatment effect among the included studies and values greater than 50% were considered to indicate high heterogeneity. A *P* value of<.05 (2-sided) was deemed statistically significant. We specified the following possible explanations for heterogeneity: study quality, specificities of the telemedicine intervention (dedicated or nondedicated app), and health care setting (ie, low- and middle-income countries vs high-income countries), owing to expected differences in health care system organization that might influence overall reperfusion times. Sensitivity analyses were predefined to evaluate the robustness of the primary outcome results. In sensitivity analyses, the effect size was examined by omitting studies individually and also excluding simultaneously studies with extreme results (highest and lowest impact). Subgroup analyses were performed to assess whether the treatment effect differed with regards to the type of technology used (dedicated vs nondedicated platform) and study quality (low vs medium to high risk of bias). Publication bias was investigated by constructing a funnel plot and plot symmetry was determined both visually and formally using Egger test.

### Ethical Considerations

This study was conducted using only aggregated, study-level data extracted from previously published sources. No individual patient-level data were accessed or analyzed, and no identifiable personal health information was used. As such, ethics approval and informed consent were not required.

## Results

### Overview

**Figure 1.**
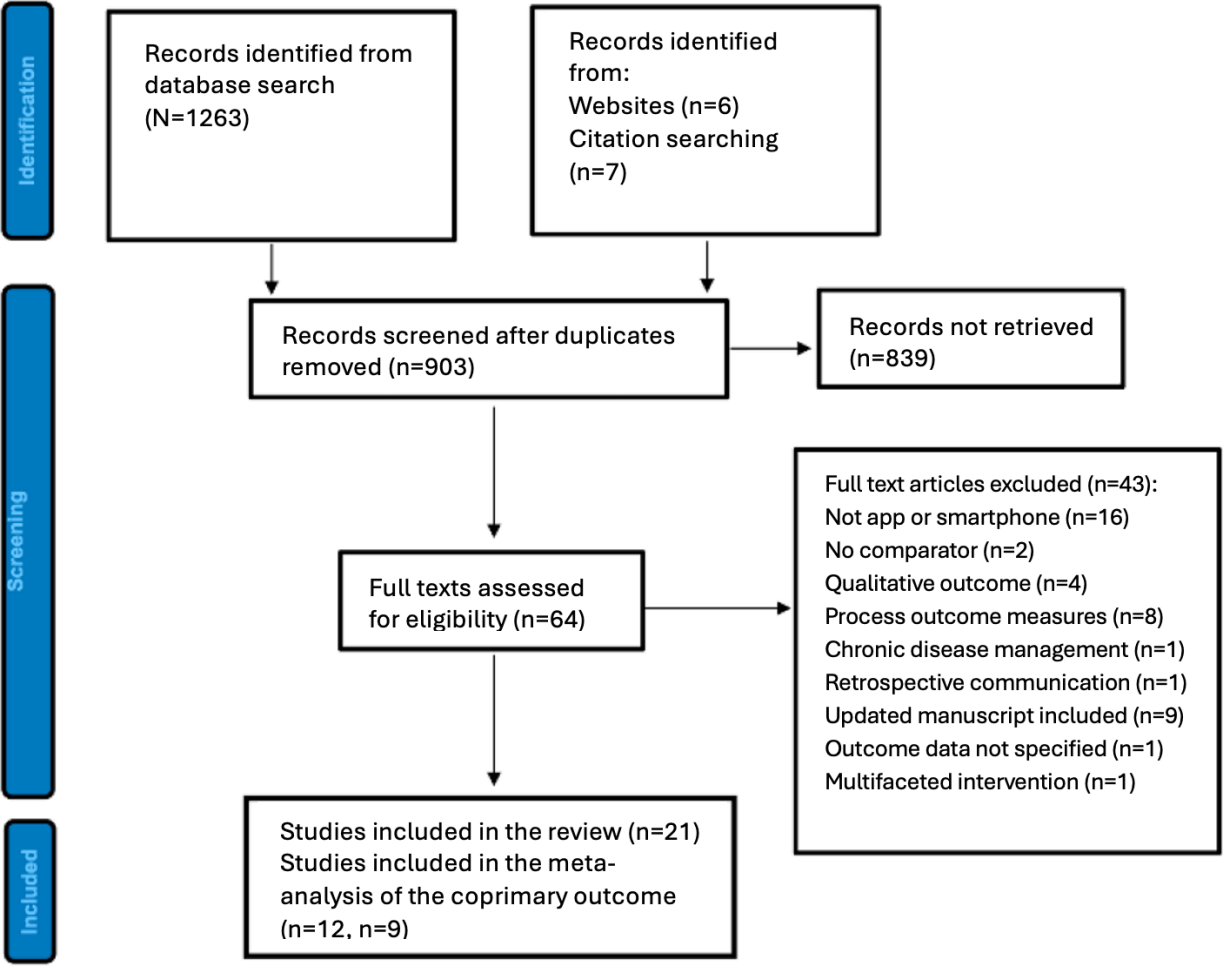
PRISMA (Preferred Reporting Items for Systematic Reviews and Meta-Analyses) flowchart of articles identified during the search process.

The search yielded 1263 results and 360 duplicates were removed. Of the remaining 903 studies, 890 were derived from databases (MEDLINE and Embase), while citations of evaluated studies and web searching (Google Scholar) identified apps provided a further 13 records for screening. Figure 1. displays the PRISMA flowchart depicting the final article inclusion. Following title and abstract screening, 839 records were removed, and the remaining 64 studies underwent full text assessment for inclusion based on the eligibility criteria. Exclusion of 43 studies was carried out with the reasons outlined (Figure 1). Thus, the final number of studies included in the review was 21, involving 3267 patients.

### Characteristics of Included Studies

The main characteristics of each included study are shown in Table 2. The selected studies were published between 2013 and 2024. A total of 18 studies were published in peer-reviewed journals and 3 were published conference abstracts [15-17]. All studies were nonrandomized in design: 14 were retrospective, involving a historical comparator group of patients treated before the implementation of a telemedicine intervention; the remaining 7 studies were prospective, quasi-experimental, and included a concurrent parallel control group in which the technology was not used [9,18-23]. The total number of participants in each study ranged from 46 to 428. The studies were conducted across 10 countries: Japan (n=4), United States (n=3), Taiwan (n=3), Canada (n=2), Australia (n=2), Argentina (n=1), Slovakia (n=1), Turkey (n=1), Egypt (n=1), and China (n=1). About 13 studies evaluated purpose-built apps, including 18 distinct platforms, while instant messaging apps (WhatsApp [n=3], LINE [n=2], Band [n=1], and WeChat (Tencent Holdings Ltd; n=2) were implemented in 8 studies [9,19,21,22,24-27]. The platforms described were commercially available from mainstream app stores. In all cases, the software was used to transmit ECGs to a dedicated chat group involving STEMI care providers, the referring practitioner, and in certain cases, the emergency physicians. The app was used in patients transferred by EMSs from the field in 14 studies, for interhospital transfer from non-PCI capable centers in 8 studies, and for patients presenting directly to the emergency department in 5 studies. In 9 studies, a combination of the type of presentation to the pPCI center was addressed. All but one [24] of the studies involving nondedicated platforms evaluated interhospital transfers of patients from non-PCI capable centers to a pPCI center.

**Table 2.** Characteristics and primary outcomes of included studies.

Study	Year	Country	Participants		Presentation			App users			Outcomes	
			(I/C)^a^	App	EMS^b^	IHT^c^	ED^d^	EMS	ED	pPCI^e^	D2B^f^ (mins)	FMC2B^g^ (mins)
Aboal et al [18]	2024	Spain	98/129	ODISEA (Biomedical Research Institute of Girona)	✓	✓	—	✓	—	✓	—	102 (SD 36)^h^ vs 117 (SD 45)^h^
Abrahim et al [28]	2023	USA	132/296	e-Bridge (General Devices LLC)	✓	—	✓	✓	✓	✓	61.2 (SD 24.3) vs 68.4 (SD 24.6)^h^	94.9 (SD 26.5) vs 100.2 (SD 28.0)^b^
Aldajani et al [29]	2024	Canada	31/84	STENOA (Stenoa Inc)	✓	—	✓	—	✓	✓	—	85.3 (SD 25.5) vs 95.5 (SD 52.2)^b^
Anai et al [15].	2024	Japan	113/130	SCUNA (Mehergen Group Holdings, Inc)	✓	—	—	✓	—	✓	60 (SD 25.1) vs 75 (SD 20)^h^	—
Arinaga et. al. [30]	2022	Japan	23/25	SCUNA	✓	—	—	✓	—	✓	49 (41‐85.5) vs 59 (38-67)^i^	72 (60.5‐107) vs 80 (63-92)^i^
Astarcioglu et al [9]	2015	Turkey	53/55	WhatsApp (Meta Platforms Inc)	—	✓	—	—	✓	✓	—	109 (SD 31) vs 130 (SD 46)^h^
Bendary et al [19]	2019	Egypt	100/100	WhatsApp	✓	✓	—	—	✓	✓	120 (SD 35) vs 157 (SD 30)^h^	—
Bladin et al [20]	2022	Australia	171/76	Pulsara (Pulsara, Inc)	✓	—	✓	✓	✓	✓	59.37 (SD 41.33) vs 77.3 (SD 61.6)^i^	—
Brouillette et al [16]	2023	Canada	31/84	STENOA	✓	—	✓	—	✓	✓	—	—
Chao et al [24]	2017	Taiwan	44/40	LINE (LY Corporation)	—	—	✓	—	✓	✓	90.4 (SD 9.8) vs 119.3 (SD 16.3)^i^	—
Cordero et al [17]	2013	Spain	177	MBEAT (ImaxDI)	✓	—	—	✓	—	✓	—	128 vs 187^h^ (p<0.01)
Dickson et al [31]	2023	USA	69/36	Pulsara (Pulsara, Inc)	✓	—	✓	✓	✓	✓	71 (SD 49) vs 91 (SD 49)^h^	—
Ibanez et al [21]	2019	Argentina	43/62	WhatsApp	✓	✓	—	✓	✓	✓	—	132 (80‐150) vs 166 (135-210)^h^
Kang et al [25]	2023	China	100/98	WeChat (Tencent Holdings Ltd)	—	✓	—	—	✓	✓	65.9 (SD 13.86) vs 98.21 (SD 21.02)^h^	98.6 (SD 18.7) vs 128.28 (SD 20.22)^h^
Kini et al [32]	2024	USA	17/43	STEMICathAid (Icahn School of Medicine at Mount Sinai)	—	✓	—	✓	✓	✓	—	86 (SD 16) vs 120 (SD 48)^h^
Kohashi et al [33]	2023	Japan	77/160	SCUNA	✓	—	—	✓	—	✓	67.7 (SD 23.6) vs 78.0 (SD 29.3)^h^	—
Liu et al [26]	2020	Taiwan	70/70	WeChat (Tencent Holdings Ltd)	—	✓	—	✓	✓	✓	60 vs 95.5^h^ (p<0.001)	132 vs 171^h^ (p<0.001)
Park et al [22]	2016	Korea	50/64	BAND (Naver Corporation/Camp Mobile)	—	✓	—	—	✓	✓	47.5 (40-56) vs 56.5 (47-69.5)^h^	102.5 (89-139) vs 129.5 (98-159)^h^
Studencan et al [34]	2018	Slovakia	178/67	STEMI (STEMI Global)	✓	—	✓	—	✓	—	—
Yu et al [27]	2019	Taiwan	51/89	LINE (LY Corporation)	—	✓	—	—	✓	✓	52.6 (SD 42.2) vs 78.4 (SD 50.6)^h^	—
Yufu et al [23]	2019	Japan	17/29	SCUNA	✓	—	—	✓	—	✓	70 (SD 26) vs 96 (SD 24)^h^	—

a I/C: intervention/control.

b EMS: emergency medical service.

c IHT: interhospital transfer.

d ED: emergency department.

e pPCI: primary percutaneous coronary intervention.

f D2B: door to balloon.

g FMC2B: first medical contact to balloon.

h *P*<.05 (significant).

i *P*≥.05 (not significant); values in parentheses: median and IQR; values with (SD): mean (SD).

### Assessment of Study Quality

Table 3 outlines the quality assessment as graded by Newcastle-Ottawa Scale. A total of 11 studies were deemed to be at low risk of bias, while 10 were considered medium to high risk of same with a median score of 6.5 (IQR 5‐7) being observed.

**Table 3.** Newcastle-Ottawa assessment scale of study quality.

Author	Year	Selection				Comparability	Outcomes			Points	RoB
		1	2	3	4	1	1	2	3		
Aboal et al [18]	2024	*	—	*	*	*	*	*	—	6	Low
Abrahim et al [28]	2024	*	*	*	*	—	*	*	—	6	M-H
Aldajani et al [29]	2024	*	*	*	*	*	*	*	—	7	Low
Anai et al [15]	2024	*	—	*	*	—	*	—	—	4	M-H
Arinaga et. al. [30]	2023	*	—	*	*	**	—	*	—	7	Low
Astarcioglu et al [9]	2015	*	*	*	*	*	—	—	—	5	M-H
Bendary et al [19]	2019	*	—	*	*	**	*	*	—	7	Low
Bladin et al [20]	2023	*	—	—	*	*	*	*	—	5	M-H
Brouillette et al [16]	2022	*	*	*	*	*	*	*	—	7	Low
Chao et al [24]	2017	*	*	*	*	*	*	*	—	7	Low
Cordero et al [17]	2014	-	*	*	*	—	—	—	—	3	M-H
Dickson et al [31]	2014	*	*	*	*	—	**	*	—	7	M-H
Ibanez et al [21]	2019	*	*	*	*	**	—	*	—	7	Low
Kang et al [25]	2024	-	*	*	*	*	—	*	—	5	M-H
Kini et al [32]	2024	*	—	*	*	—	*	*	—	5	M-H
Kohashi et al [33]	2023	*	*	*	*	**	*	*	*	9	Low
Liu et al [26]	2020	*	*	*	*	*	*	*	—	7	Low
Park et al [22]	2016	*	—	*	*	—	—	—	—	3	M-H
Studencan et al [34]	2018	*	—	*	*	*	*	*	—	6	Low
Yu et al [27]	2019	*	—	*	—	*	—	—	*	4	M-H
Yufu et al [23]	2019	*	*	*	*	**	—	*	*	8	Low

a RoB: risk of bias.

b M-H: medium to high.

### Outcomes

#### Primary Outcome

With respect to the primary outcome, all but one of the included studies evaluated the association of a digital telemedicine strategy with reperfusion times, including door to balloon, FMC2B, or total ischemic time. The remaining study investigated “false activations” [16] exclusively within a similar patient cohort from another included study [29]. Time target definitions varied across studies and were standardized according to our prespecified definitions as follows: door-to-device time was used in one study in reference to “the interval from the initial presentation at the referring hospital until the first balloon inflation or use of another device to achieve reperfusion” [32] and thus, we labeled this as the FMC2B time. Similarly, “first medical contact to wire” [25] or “device” [22,29] was considered the FMC2B, as was “ECG diagnosis to wire crossing.” Regarding meta-analysis, complete data including both the mean and SD for the treatment and control groups was documented in 13 studies. The individual SDs were not reported in 2 studies [15,31] and were therefore estimated from the mean difference and provided *P* value using validated methods [14]. Furthermore, the median value alone were reported in 5 studies; one author provided the necessary data upon request [33] and mean and SD values were estimated, accordingly for the remaining 4 [20-22,30]. A further study was excluded from quantitative data synthesis [17] as it did not report the number of participants in both the treatment and control groups.

**Figure 2.**
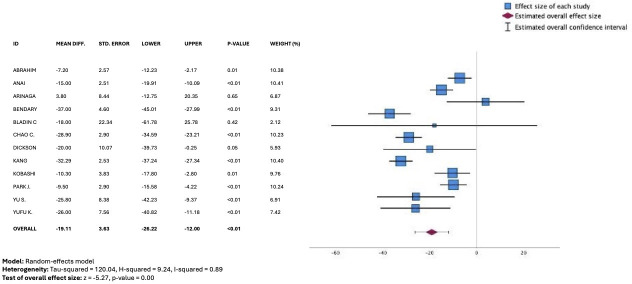
Forest plot from meta-analysis of unstandardized mean difference in door-to-balloon times (minutes) comparing telemedicine strategies versus usual care [15,19,20,22-25,27,28,30,31,33].

**Figure 3.**
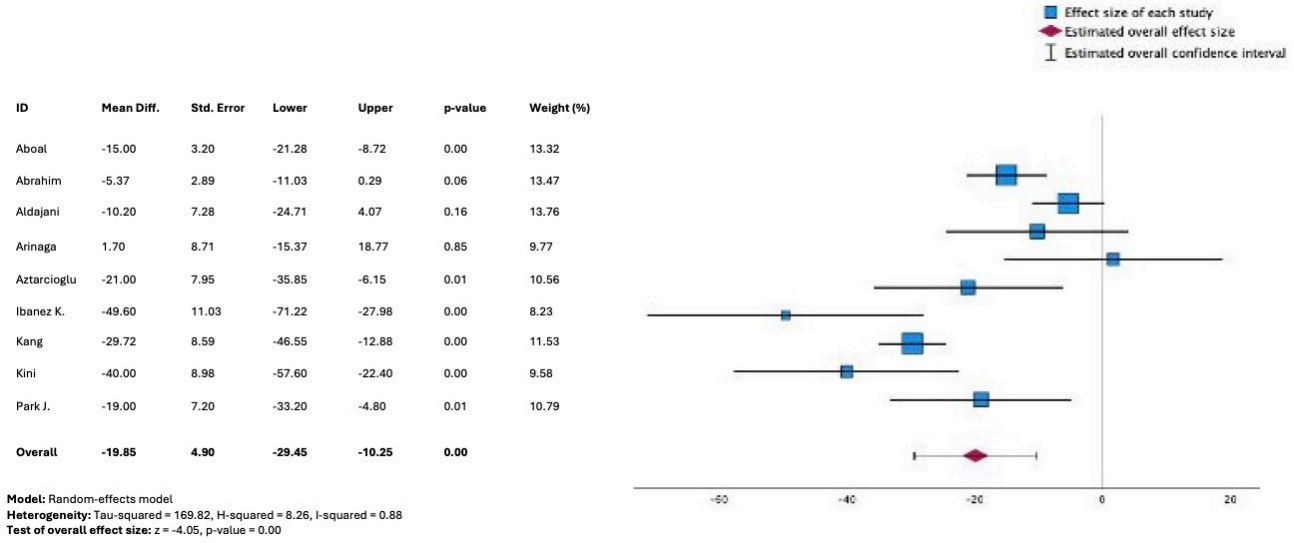
Forest plot from meta-analysis of unstandardized mean difference in first medical contact-to-balloon times (minutes) comparing telemedicine strategies versus usual care [9,11,21,22,25,28-30,32].

Differences in D2B times versus usual care were reported in 13 studies with sufficient data available from 12 studies to conduct meta-analysis, involving 1977 patients. There was a significant difference in D2B times observed, favoring a smartphone app–based intervention versus usual care (unstandardized mean difference, −19.11 mins, 95% CI −26.22 to −12.00); *P*<.01) (Figure 2 [15,19,20,22-25,27,28,30,31,33]), while substantial statistical heterogeneity was evident (*I*^2^=89%). FMC2B times were reported in 11 studies overall, with 9 included in the meta-analysis (n=1888). A similar treatment effect was evident, favoring the intervention group (unstandardized mean difference, −19.85 mins, 95% CI −29.45 to –20.25; *P*=.01; Figure 3 [9,11,21,22,25,28-30,32]), with similar degrees of statistical heterogeneity found (*I*^2^=88%).

#### Secondary Outcomes

**Figure 4.**
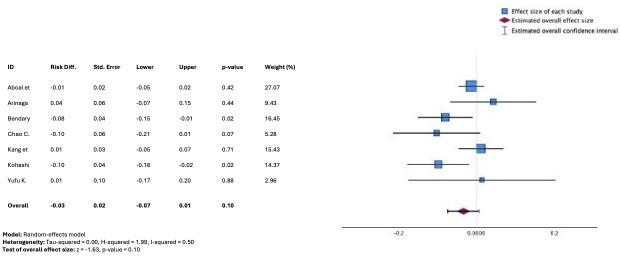
Forest plot from meta-analysis of risk difference in in- hospital or 30-day mortality comparing telemedicine strategies versus usual care [11,19,23-25,30,33].

Mortality was evaluated in 8 studies, including 1711 patients, half of these reported in-hospital mortality [18,19,23,25], 30-day mortality in 3 [24,30,33], and one-year mortality in the remaining study [15]. For meta-analysis, the study involving one-year mortality was excluded, resulting in a total of 7 publications included in the pooled analysis. There was no significant effect observed with respect to short-term mortality associated with a smartphone-based strategy versus usual care (Risk difference −0.03, 95% CI −0.07 to 0.01); *P*=.10; Figure 4 [11,19,23-25,30,33]).

Furthermore, 7 studies evaluated the impact of an app-based coordination pathways on false or inappropriate activations of the cardiac cath lab. Due to substantial variability among studies in defining false activations and comparator groups, however, meta-analysis was not feasible. Astarcioglu et al reported false positive STEMI activations in 5 out of 55 cases in the intervention group with WhatsApp versus none in the control group [9]. Brouillette et al [16] defined false activations as cancellations of cath lab activations prior to any procedure and demonstrated a reduction in false activations with use of the STENOA platform, decreasing from 9.4% to 6.4%, a 31% relative reduction (*P*<.05). Furthermore, the use of STEMICATHAID (Icahn School of Medicine at Mount Sinai) facilitated preactivation screening, rejecting 45 out of 111 activations prior to cath lab mobilization, however, no false activation data was available for comparison in the control period [32]. Dickson et al [31] observed improved resource use with a reduction in PCI procedures following implementation of the Pulsara (Pulsara, Inc) app (from 75% to 64%), representing an 11% absolute reduction. Meanwhile, there were fewer misdiagnoses of acute coronary syndrome in the group using the ODISEA (Biomedical Research Institute of Girona) app compared to controls (9.5% vs 17.1%, *P*=.004) [18]. In addition, another included study noted fewer inappropriate activations in the arm assigned to the SCUNA mobile ECG transmission system (13/36 vs 20/45) [30].

Finally, the proportion of patients meeting D2B or FMC2B times of <90 and <120 minutes were reported in 6 [9,20,23,24,31,34] and 4 studies [18,22,32,34], respectively. A pooled analysis of these outcomes combined demonstrated a higher likelihood of patients treated in the intervention arm of meeting guideline directed reperfusion times compared to those assigned to usual care (OR 3.18, 95% CI 1.59‐6.35; *P*<.001; *I*^2^=0.77; Figure S1 in Multimedia Appendix 1).

#### Publication Bias

Publication bias was assessed using funnel plots for the primary outcomes of D2B and FMC2B times. Visual inspection of the funnel plots revealed asymmetry for D2B time, suggesting potential publication bias, whereas minimal asymmetry was observed for FMC2B time. This was further supported by Egger test, which indicated significant publication bias for D2B time (*P*=.01) but not for FMC2B time (*P*=.5; Figures S2 and S3 in Multimedia Appendix 2 and Multimedia Appendix 3).

#### Sensitivity and Subgroup Analysis

Sensitivity analysis using the leave-one-out method, where individual studies were sequentially excluded, demonstrated no significant impact on the overall treatment effect for D2B or FMC2B time, confirming the robustness of the findings. Subgroup analysis based on geographical setting (low- and middle-income countries vs high-income countries) revealed a greater reduction in D2B time in studies conducted in low-income settings (mean difference −31.48 mins, 95% CI −24.86 to −28.11) compared to those in high-income settings (mean difference −11.26 mins, 95% CI −15.18 to −7.34; Figure S4 in Multimedia Appendix 4). However, this trend was not as pronounced for FMC2B time (low-income −31.02 mins, 95% CI −42.94 to −19.11 vs high-income −13.86 mins, 95% CI −23.64 to −4.07). Further subgroup analyses stratified according to the use of dedicated versus nondedicated mobile apps, risk of bias (high vs low), and study sample size (above vs below the median) showed no statistically significant differences between subgroups.

To explore potential sources of heterogeneity, meta-regression analysis was performed. For D2B time, geographical setting accounted for nearly all the observed heterogeneity (*R*²=100%), suggesting a strong influence of regional health care disparities. (Figure S2 in Multimedia Appendix 3). In contrast, for FMC2B time, geographical setting explained only a moderate proportion of heterogeneity (*R*²=31.4%). Other factors, including sample size, use of dedicated versus nondedicated apps, and study quality, had minimal influence on heterogeneity for both D2B and FMC2B times.

## Discussion

### Principal Findings

In this comprehensive systematic review and meta-analysis, we synthesized evidence from 21 studies, including 3267 patients, which evaluated smartphone apps implemented within acute STEMI care pathways. The included studies assessed the impact of these digital platforms on key reperfusion metrics and clinical outcomes. Our findings suggest that an average reduction in reperfusion times of up to 20 minutes might be achieved with the integration of smartphone apps in STEMI care pathways, with the potential for a larger proportion of patients achieving guideline directed target times (D2B <90 mins and FMC2B <120 mins) versus usual care. mHealth technologies have emerged as transformative tools in acute care settings, with up to 40% of emergency-focused mHealth solutions prioritizing rapid communication and coordination functions [8]. Immediate and accurate sharing of patient clinical and treatment information with the receiving hospital is essential in acute STEMI and a single communication channel via smartphone technology used both within the hospital and by prehospital personnel strengthens the integration of treatment advances that cover the community and medical care settings alike [20].

To our knowledge, this is the first meta-analysis specifically examining the effectiveness of dedicated smartphone-based apps within STEMI care networks. Previous systematic reviews and meta-analyses have demonstrated improvements in reperfusion times and mortality with telemedicine-based strategies, as well as digital transmission of the prehospital electrocardiogram in acute STEMI care [6,35]. However, none have exclusively addressed interventions incorporating the broader functionalities provided by smartphone apps, such as coordinated alerts, real-time geolocation, automated data collection, and integrated communication systems among multidisciplinary STEMI teams, as we have described. Since telemedicine interventions are rarely effective in isolation and instead function best as part of a multifaceted strategy integrating prehospital and in-hospital services, it stands to reason that smartphone apps offering these multiple functionalities could further streamline workflows and enhance the efficiency of STEMI care processes.

Our review further identified multiple studies reporting a significant reduction in inappropriate cath lab activations following the implementation of an app-based strategy. Inappropriate activation of the cath lab represents a substantial burden to health care systems with disruption of clinical workflows, prompting unnecessary mobilization of on-call teams, while potentially causing unnecessary harm to patients [36]. The capacity to integrate real-time ECG assessment with structured case adjudication, supported by comprehensive clinical data, may enhance diagnostic accuracy and communication among care teams, thereby reducing rates of false-positive activations.

Notably, we found no significant difference in short-term mortality between the intervention and control arms which is in contrast with the findings of a recent meta-analysis of studies evaluating the impact of digital prehospital ECG transmission in acute STEMI [35]. Therein, the authors observed a significant reduction in all-cause mortality (hazard ratio 0.53, 0.40‐0.69), which accompanied a greater reduction in mean door and FMC2B times than we found (mean difference −33.31 mins, 95% CI −50.47 to −16.16) However, the substantial weighting of included studies (>50%) which reported long-term mortality [37], in addition to the inclusion of larger studies with higher event rates, somewhat limits comparability to our findings. Furthermore, differences in baseline and procedural characteristics or study design may have contributed to the differences observed. They further noted substantial statistical heterogeneity (>99%) with respect to reperfusion times while demonstrating a significantly higher impact on subgroup analysis between interventions that integrated both prehospital digital ECG transmission and early cath lab activation versus ECG alone. This may be reflective of the earlier study period of their included studies, during which time STEMI care networks were comparatively rudimentary to those evaluated in our analysis, with a greater potential to yield marginal benefits from organizational improvements. Likewise, our meta-regression analysis revealed that geographical location (ie, low- vs high-income countries) was the strongest factor contributing to the observed heterogeneity in terms of D2B times. Similarly, health care systems across low-income countries may have fewer care coordination processes in place and may therefore expect to achieve a greater benefit from quality improvement interventions. Since all studies from low- and middle-income countries used nondedicated apps, it remains unclear whether the additional functionalities of dedicated software could lead to further improvements, and future research is needed to evaluate their use in these settings.

On the other hand, the influence of geographical location was less pronounced in terms of FMC2B times. A possible explanation for this disparity is that system-level factors—such as prehospital care infrastructure and EMSs efficiency—may have a greater impact on FMC2B time, whereas in-hospital workflow efficiency primarily affects D2B time. In low-income settings, delays in prehospital activation and interfacility transport [38] may have contributed to the persistence of FMC2B disparities despite significant reductions in D2B time. However, most included studies did not report data on average transport times or distances to pPCI centers, preventing meta-regression analysis from assessing whether these factors contributed to the observed heterogeneity.

Nearly half of the included studies were assessed as having moderate to high risk of bias, primarily attributed to insufficient reporting or adjustment for baseline characteristic disparities. However, subgroup analyses comparing studies with a high and low risk of bias revealed no statistically significant differences in treatment effects across outcomes, suggesting minimal influence of bias on pooled results.

We also identified a number of important distinctions between dedicated and nondedicated apps in the context of acute STEMI care (Table 4). Concerning the point of care, the functions of instant messaging apps are largely confined to the facilitation of rapid ECG transmission and clinical particulars via short messaging. On the other hand, dedicated apps allow for the seamless input of various data points to designated fields while enabling custom notifications and alert systems, which can confirm the acknowledgment of each team member’s participation and readiness, automatically signal the presence of high-risk clinical features and with GPS integration, can prompt consideration of alternative treatment strategies if expected patient arrival times exceed guideline-directed targets. Moreover, the majority of dedicated apps identified boast functions beyond the point of care with automated storage of quality assurance data which can streamline clinical auditing and ultimately lessen the significant administrative burden associated with such processes. Furthermore, dedicated platforms can be structured appropriately to ensure that the handling of protected health information data is strictly compliant with local data security regulations. This is especially important for patients, as research indicates they place high value on robust data privacy and security measures in mHealth apps. Therefore, dedicated platforms that adhere to regulatory frameworks are essential to ensure compliance, build trust, and overcome adoption barriers [39]. In contrast, the use of nondedicated apps in this context raises concerns about their appropriateness.

**Table 4.** Key functions enabled by different mobile app types.

Features	Nondedicated	Dedicated
Cloud-based ECG^a^ transmission	✓	✓
Real time communication	✓	✓
Automatic notification of provider readiness		✓
Real time treatment target tracking**^b^**		✓
Automated data recording and reporting		✓
GPS location enabled		✓
Security and compliance		✓

a ECG: electrocardiogram.

b Automatic notification when estimated first medical contact-to-balloon time exceeds target with recommendation to alter treatment course ie, fibrinolytic therapy.

### Limitations

All of the included studies were observational in design and mostly comprised of relatively small sample sizes. Therefore, many studies were underpowered to establish definitive conclusions regarding the impact of smartphone-based interventions on critical clinical outcomes, such as mortality. Nevertheless, the clinical implications of our findings remain important, given existing evidence linking longer D2B times to increased short-term mortality. A pooled analysis involving over 70,000 patients, for example, demonstrated significantly higher short-term mortality among patients with D2B times exceeding 90 minutes (OR 1.39, 95% CI 1.19‐1.62) [40]. Similarly, an individual patient data analysis comprising more than 4000 patients found consistent results, even after adjusting for multiple potential confounders [3]. A further limitation of this study is the reliance on statistical imputation for a number of outcome variables, as only the median and IQR values were provided in the original reports. This approach may have introduced bias and limited the precision of our analysis, owing to uncertainties regarding the underlying distribution of the data [41]. In addition, the lack of distributional data in most studies in terms of different patient presentation modes (ie, direct ED, EMS, or interhospital transfer) poses a limitation. The impact of the app on outcomes across these modes is not yet clear, and further investigation is needed to determine whether the use of smartphone apps has a differential effect based on the mode of presentation.

### Conclusion

Smartphone apps are playing an increasingly transformative role in emergency care, offering the potential to enhance processes and, ultimately, improve clinical outcomes. By consolidating critical functionalities into a single platform, these apps address the systemic fragmentation between pre-hospital and in-hospital care, facilitating more coordinated workflows. Our analysis highlights a reduction in reperfusion times with the integration of these systems; however, larger prospective studies are needed to further explore and validate these findings.

## Supplementary material

10.2196/66605 Multimedia Appendix 1 Achieving door to balloon time of <90 minutes or first medical contact to balloon time of <120 minutes in patients submitted to intervention versus usual care.

10.2196/66605 Multimedia Appendix 2 Funnel plot assessing publication bias for the primary endpoint of door to balloon times.

10.2196/66605 Multimedia Appendix 3 Funnel plot assessing publication bias for publication bias with respect to the outcome of first medical contact to balloon times.

10.2196/66605 Multimedia Appendix 4 Subgroup analysis of primary endpoint door to balloon times stratified according to health care setting (1: low- or middle-income countries versus 2: high-income countries).

10.2196/66605 Checklist 1 PRISMA (Preferred Reporting Items for Systematic Reviews and Meta-Analyses checklist.

## Abbreviations

Cath lab: catheterization laboratory
D2B: door to balloon
ECG: electrocardiogram
ED: emergency department
EMS: emergency medical service
FMC2B: first medical contact to balloon time
mHealth: mobile health
ODISEA: Biomedical Research Institute of Girona
OR: odds ratio
PCI: percutaneous coronary intervention
PICO: populations, people, patient, or problem, intervention, comparison, and outcome
pPCI: primary percutaneous coronary intervention
PRISMA: Preferred Reporting Items for Systematic Reviews and Meta-Analyses
PROSPERO: International Prospective Register of Systematic Reviews
STEMI: ST-segment elevation myocardial infarction
