# Supplementary figures and images for: Impact of Smartphone Apps on Reperfusion Times and Clinical Outcomes in Acute ST-Segment Elevation Myocardial Infarction: Systematic Review and Meta-Analysis
Source: JMIR Mhealth Uhealth. 2025 Aug 25;13:e66605. doi: 10.2196/66605 (PMC12377873; doi:10.2196/66605)

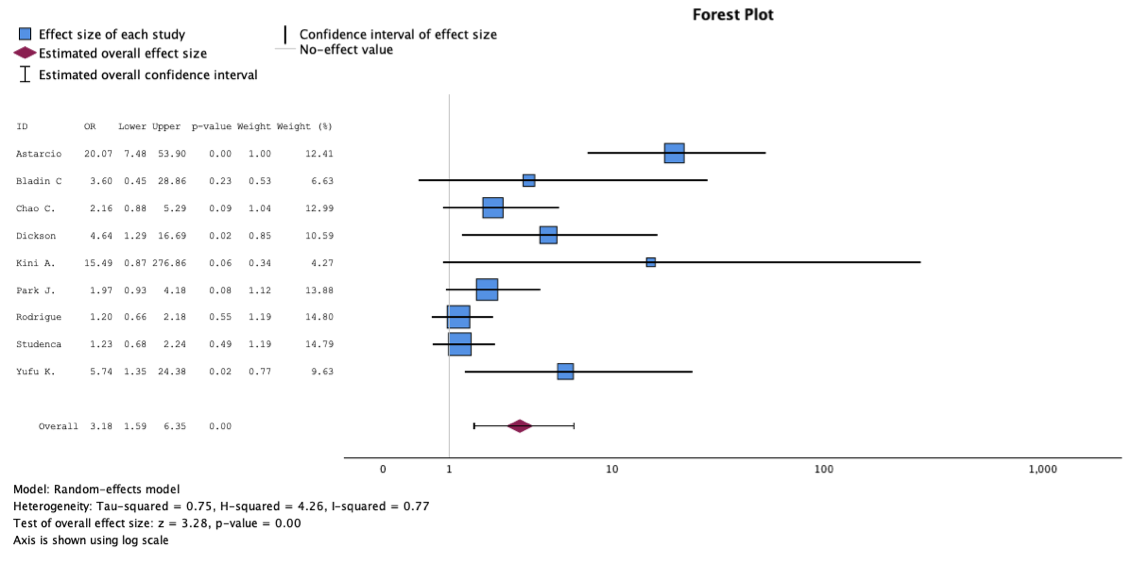

Supplement: Multimedia Appendix 1 [file mhealth-v13-e66605-s001.png]

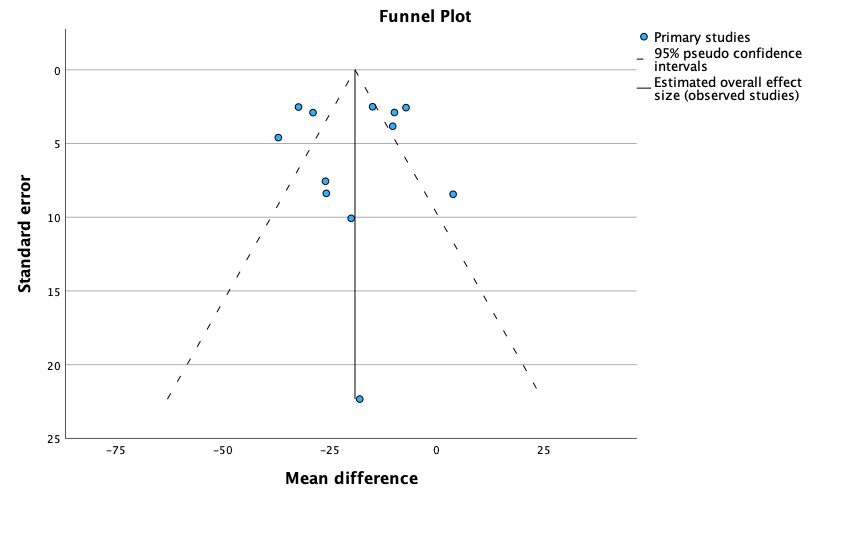

Supplement: Multimedia Appendix 2 [file mhealth-v13-e66605-s002.png]

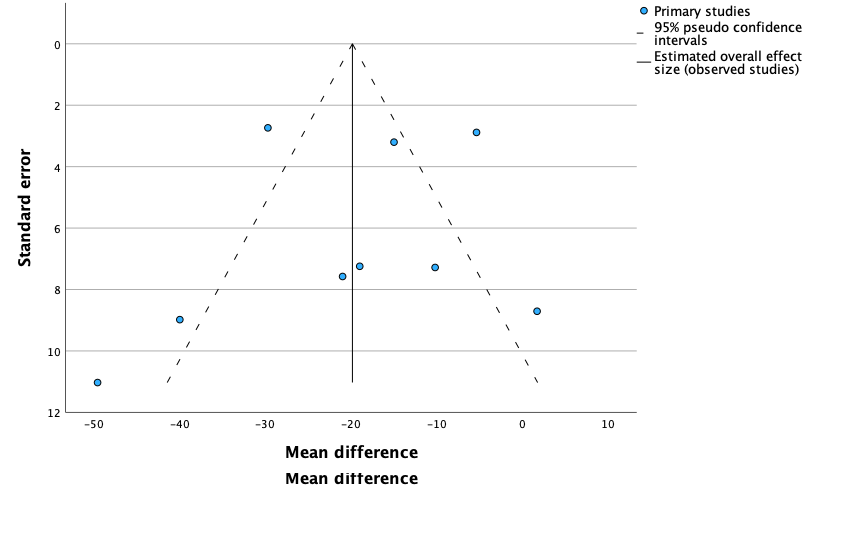

Supplement: Multimedia Appendix 3 [file mhealth-v13-e66605-s003.png]

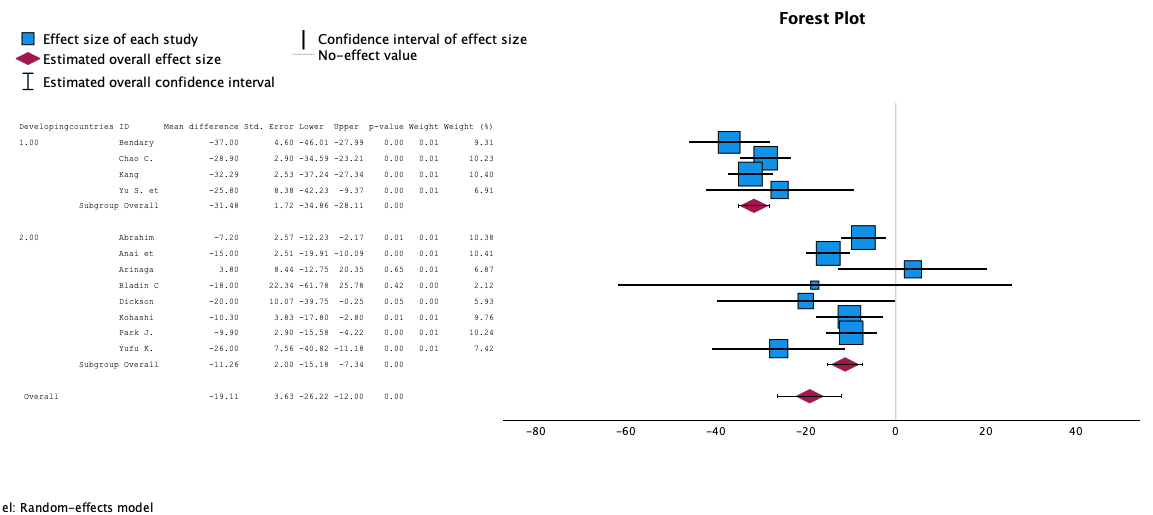

Supplement: Multimedia Appendix 4 [file mhealth-v13-e66605-s004.png]
